# High Prevalence of Multidrug-Resistant Enterobacteriaceae Uropathogens Among Outpatients in Rural Southwestern Uganda

**DOI:** 10.7759/cureus.78094

**Published:** 2025-01-27

**Authors:** Barbra Tuhamize, Deusdedit Tusubira, Charles Masembe, Pascal O Bessong, Frederick Byarugaba, Joel Bazira

**Affiliations:** 1 Department of Microbiology, Mbarara University of Science and Technology, Mbarara, UGA; 2 Department of Biochemistry, Mbarara University of Science and Technology, Mbarara, UGA; 3 Department of Zoology, Entomology and Fisheries Sciences, Makerere University, Kampala, UGA; 4 Center for Global Health Equity, University of Virginia, Charlottesville, USA; 5 School of Health Sciences, University of KwaZulu-Natal, Durban, ZAF; 6 South African Medical Research Council (SAMRC) University of Venda Antimicrobial Resistance and Global Health Research Unit, University of Venda, Thohoyandou, ZAF

**Keywords:** antimicrobial resistance (amr), multi-drug resistance (mdr), pan drug resistance (pdr), rural south western uganda, urinary tract infections (utis), uropathogenic enterobacteriaceae

## Abstract

Background

Urinary tract infections (UTIs) are among the most common bacterial infections globally, with Enterobacteriaceae, particularly *Escherichia coli* and *Klebsiella pneumoniae*, being the primary causative agents. The rising prevalence of antimicrobial resistance (AMR) in these pathogens has complicated treatment, posing a significant public health challenge. In resource-limited settings like Southwestern Uganda, limited surveillance data and inappropriate antimicrobial use exacerbate the AMR crisis. Understanding the antibiotic susceptibility profiles of uropathogenic Enterobacteriaceae is crucial to inform empirical treatment and guide local antimicrobial stewardship efforts. This study investigated the antimicrobial susceptibility patterns of Enterobacteriaceae isolated from UTI patients to commonly used antibiotics.

Methods

A hospital-based cross-sectional study was carried out in Bwizibwera Health Center IV and Rubaya Health Center III in rural Southwestern Uganda, targeting 455 individuals who were confirmed to have Enterobacteriaceae infections. These participants were drawn from a pool of 2,371 patients presenting with urinary tract infections (UTIs) at the two health centers. Identification of Enterobacteriaceae was performed using a range of biochemical tests, and antimicrobial susceptibility patterns were assessed using the Kirby-Bauer disk diffusion technique with specific antibiotics. The data were analyzed and summarized as frequencies and percentages, with results presented in tables and graphs.

Results

Out of the 2371 participants, 455 (19.2%, CI 17.6 - 20.8) tested positive for uropathogenic Enterobacteriaceae. Out of the 455, the majority were females (398/455, 87.5%). The mean age of the study participants was 41.6 (SD 19.7) years. Each of the 455 study participants had a single uropathogen. Organisms were highly resistant to tetracycline with a proportion of 89.5% (407/455) and least resistant to meropenem 76 out of 455 (16.7%) and ceftriaxone 79 out of 455 (17.9%). Multidrug resistance (MDR) was observed in 114 out of 455 (25.05%) of the organisms, whereas 10.77% (49/455) of the organisms exhibited pan-drug resistance (PDR). In addition, MDR was highest in isolates from females, and MDR and PDR were highest among individuals 60 years and above.

Conclusion

Our study revealed a high prevalence of Enterobacteriaceae uropathogens isolated in rural South Western Uganda predominantly affecting women. The organisms showed the highest resistance to tetracycline and varying degrees of antibiotic resistance (MDR, extensive drug resistance (XDR), and PDR), including carbapenem-resistant strains of *E. coli*, *Klebsiella*, and *Enterobacter* spp., posing a significant public health threat. These findings highlight the urgent need for revised antibiotic prescription practices, strengthened antibiotic stewardship, and continuous surveillance of uropathogen susceptibility patterns to guide effective empirical treatment strategies.

## Introduction

Urinary tract infections (UTIs) are among the most common bacterial infections globally, affecting millions of individuals annually and posing significant healthcare challenges, particularly in resource-limited settings. Enterobacteriaceae, a family of Gram-negative bacteria, are the predominant causative agents of UTIs, with *Escherichia coli* and *Klebsiella pneumoniae* being the most frequently isolated pathogens [1]. The increasing prevalence of antimicrobial resistance (AMR) within this group is a critical public health concern, as it complicates treatment, prolongs hospital stays, and escalates healthcare costs [2].

In sub-Saharan Africa, including Uganda, resistance to commonly used antibiotics, such as β-lactams, fluoroquinolones, and aminoglycosides, is on the rise [3] giving rise to multidrug resistant (MDR), extensive-drug-resistant (XDR), and pan-drug-resistant (PDR) organisms. This trend is driven by factors such as the inappropriate use of antibiotics, lack of robust antimicrobial stewardship programs, and limited access to diagnostic tools [4]. Consequently, empirical treatment guidelines are often outdated, leading to suboptimal patient outcomes.

Previous studies underscore the importance of continuous surveillance of antimicrobial susceptibility patterns to inform local and regional treatment guidelines [5]. However, there is a paucity of updated data on the antimicrobial susceptibility profiles of Enterobacteriaceae uropathogens in rural Southwestern Uganda, a region characterized by a high burden of UTIs. This study aimed to investigate the susceptibility patterns to commonly used antibiotics of Enterobacteriaceae isolated from outpatient UTI patients. The findings will provide critical insights for the revision of empirical treatment guidelines, enhancing the management of UTIs in this region.

## Materials and methods

This was a cross-sectional, hospital-based study carried out at two healthcare facilities: Bwizibwera Health Center IV and Rubaya Health Center III from January 2024 to August 2024. These facilities serve diverse populations of over 120,000 people in rural and semi-urban areas, providing an ideal setting for assessing the antimicrobial susceptibility profiles of Enterobacteriaceae associated with urinary tract infections (UTIs).

Study population and sampling

The study screened a total of 2,371 outpatients presenting with symptoms suggestive of UTIs. Inclusion criteria were individuals with clinical signs of UTIs who consented to provide urine samples for laboratory analysis. From these, 455 participants were confirmed to have Enterobacteriaceae uropathogens through culture and biochemical identification. Participants were selected consecutively to ensure a representative sample of patients with UTIs during the study period.

Laboratory procedures

Sample Collection and Processing

Midstream urine samples were collected from each participant using sterile containers. Samples were refrigerated at 2 - 8 °C to preserve the specimen integrity, these were then transported to the laboratory within 4 hours for immediate processing to prevent contamination or overgrowth of non-pathogenic organisms.

Bacterial Identification

Urine samples were inoculated on MacConkey agar, blood agar, and chocolate agar and incubated at 37 °C for 24 - 48 hours. Colonies showing growth were further subjected to a series of biochemical tests, including, Oxidase, Triple Sugar Iron (TSI), Indole, Citrate, Urease, Motility, to confirm the presence of Enterobacteriaceae as described by Zakrzewski et al. [6]. 

Antimicrobial Susceptibility Testing

Antimicrobial susceptibility testing (AST) was performed using the Kirby-Bauer disk diffusion method [7] in accordance with the Clinical and Laboratory Standards Institute (CLSI) guidelines. A standardized bacterial suspension, equivalent to 0.5 McFarland turbidity, was inoculated on Mueller-Hinton agar. Antibiotic discs: amoxicillin (AX), amoxicillin and clavulanic acid (AMC), ciprofloxacin (CIP), tetracycline (TET), ceftriaxone (CRO), cefuroxime (CXM), meropenem (MEM), ertapenem (ETP) were placed on the inoculated agar plates, followed by incubation at 37 °C for 18 - 24 hours. Zones of inhibition were measured in millimeters and interpreted using CLSI breakpoints of 2019.

Quality Control

To ensure accuracy, *Escherichia coli *ATCC 25922 and *Klebsiella pneumoniae *ATCC 700603, and *Escherichia coli *ATCC 35218 were used as control strains for susceptibility testing. Each batch of media and reagents was checked for sterility and performance before use.

Data Analysis

Data were entered into Excel (Microsoft Corporation, Redmond, USA), and analyzed using SPSS version 26 (IBM Corp., Armonk, USA) to determine the prevalence of antimicrobial resistance (AMR) patterns among Enterobacteriaceae uropathogens. Frequencies and percentages were calculated for categorical variables. Measures of central tendency and dispersion for continuous variables and Chi squares to compare proportions of categories were computed, and significance at p < 0.05.

Ethical Considerations

The Research Ethics Committees of Bishop Stuart University (BSU) #BSU-REC-2023-156 and the Uganda National Council of Science and Technology issued ethical approval #HS3361ES. Written informed consent was obtained from all participants. Confidentiality and anonymity were maintained throughout the study. Consent was obtained or waived by all participants in this study.

## Results

**Figure 1:**
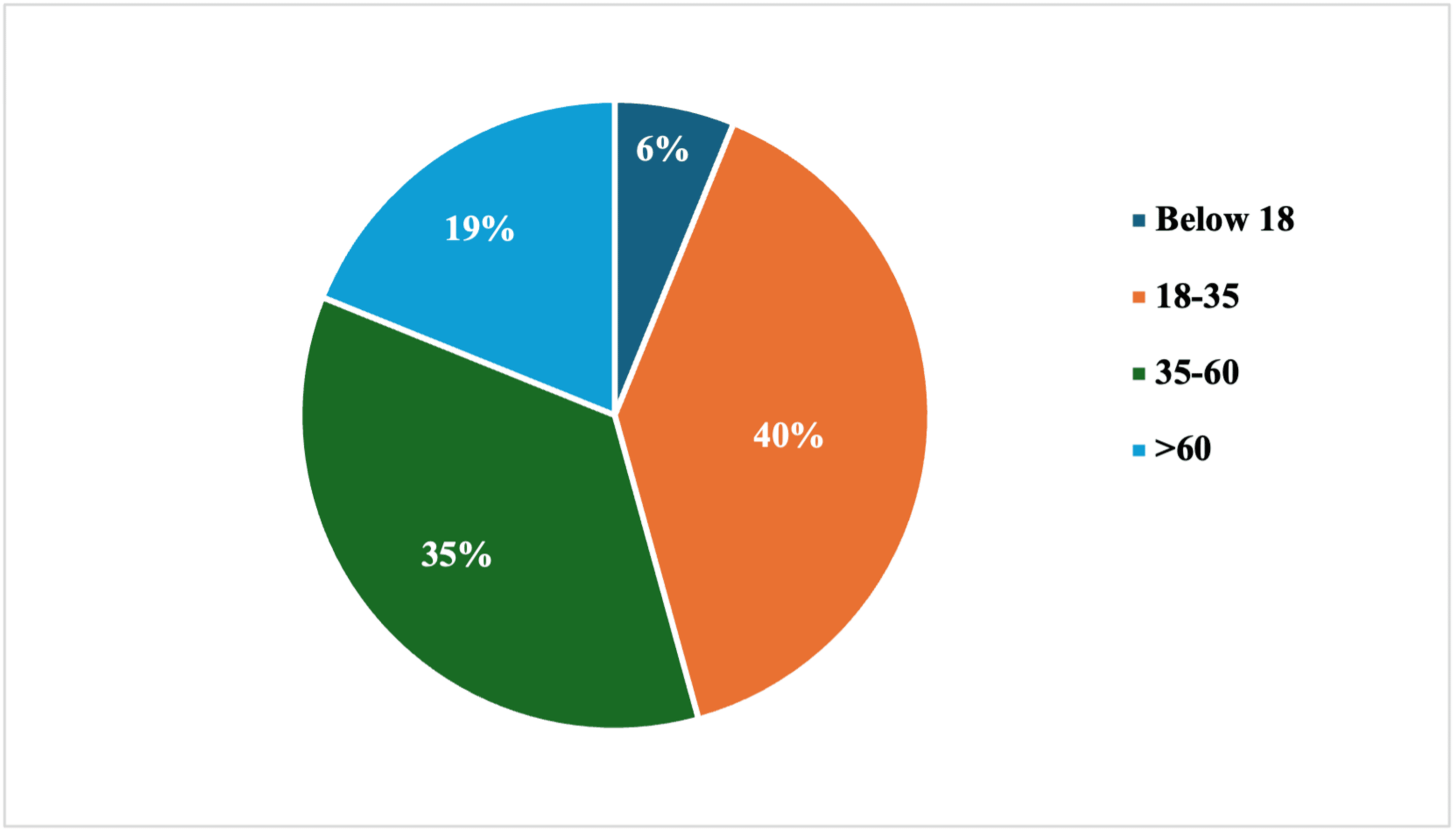
Age categories of study participants. The majority of the participants were in the age category of 18 - 35 years.

Females were the most predominant of the participants 398/455 (87.5%). The mean age of the study participants was 41.6 (SD 19.7) years, with a range of 7 to 100 years. The majority of the participants were in the age category of 18 - 35 years (Figure 1).

Prevalence of UTIs

The prevalence of UTI due to Enterobacteriaceae was 455 out of 2371 (19.2%, 95% CI 17.6 - 20.8), with the proportions of men and women as 57 (2.4%) and 398 (16.8%) out of 2371, respectively. As reported earlier [8], the most prevalent uropathogenic Enterobacteriaceae were *Escherichia coli *and *Klebsiella species*. Each of the 455 study participants had a single uropathogen. 

Organism Distribution

*E. coli *was the most prevalent in the females. Among the males, *E. coli *and* Klebsiella *species had the same distribution. Overall, the uropathogens were proportionately distributed among genders (p=0.109) and age (0.93) categories (Table 1).

**Table 1:** Organism distribution by gender and age groups P-values were calculated using chi-square test, significance at p < 0.05

	Organisms						P-value
	*Citrobacter *spp.	E. coli	Enterobacter agglomerans	Enterobacter cloacae	*Klebsiella *spp.	*Proteus *spp.	
Gender							
Female	1	188	25	10	173	1	0.109
Male	0	26	2	1	26	2	
Age categories (in years)							
Below 18	0	14	1	2	11	0	0.93
18-34	1	87	12	4	74	2	
35-60	0	76	8	3	73	1	
Above 60	0	37	6	2	41	0	

Antibiotic Resistance Patterns by Organisms

Organisms were mostly resistant to tetracycline with a proportion of 89.5% (407/455). Meropenem 76 out of 455 (16.7%) and ceftriaxone 79 out of 455 (17.9%) showed the least resistance as summarized in Table 2.

**Table 2:** Antibiotic resistance patterns by organisms. AX: amoxicillin; AMC: amoxicillin and clavulanic acid; CIP: ciprofloxacin; TET: tetracycline; CRO: ceftriaxone; CXM: cefuroxime; MEM: meropenem; ETP: ertapenem

	Antibiotics							
Organisms	AX	AMC	CIP	TET	CRO	CXM	MEM	ETP
*Citrobacter *spp.	1	1	1	1	0	0	0	0
E. coli	147	153	42	195	29	37	30	35
Enterobacter agglomerans	16	16	4	27	3	3	2	2
Enterobacter cloacae	8	10	5	6	3	4	2	4
*Klebsiella *spp.	154	155	59	175	44	53	42	51
*Proteus *spp.	1	1	0	3	0	0	0	0
TOTAL (%)	327 (71.9)	336 (73.8)	111 (24.4)	407 (89.5)	79 (17.9)	97 (21.3)	76 (16.7)	92 (20.2)

Table 3 represents the categorization of various antibiotic resistance by the bacterial isolates either as multidrug resistance (MDR), extensive drug resistance (XDR), or pan-drug resistance (PDR). The highest MDR was observed for *Klebsiella *spp. 53 (26.63%), followed by *Escherichia coli *55 (25.70%). *Enterobacter cloacae* showed the highest level of XDR resistance 2 (18.18%), whereas *Klebsiella *spp.* *showed the highest level of PDR. *Proteus *spp. did not show any MDR, XDR, or PDR. 

**Table 3:** Categorization of bacterial isolates according to antibiotic resistance MDR (multidrug resistance): non-susceptible to ≥ 1 agent in ≥ 3 antimicrobial categories; XDR (extensive drug resistance): non-susceptible to ≥ 1 agent in all but ≤ 2 categories; PDR (pan-drug resistance): non-susceptible to all antimicrobial categories. Antimicrobial categories included: penicillins (amoxicillin, amoxiclav), fluoroquinolones (ciprofloxacin), tetracyclines (tetracycline), cephalosporins (ceftriaxone and cefixime), carbapenems (imipenem and meropenem)

Bacterial isolates	Category of antibiotic resistance		
	MDR n (%)	XDR n (%)	PDR n (%)
*Citrobacter *spp. (n= 1)	1 (100)	0 (0.00)	0 (0.00)
*E. coli* (n= 214)	55 (25.70)	7 (3.27)	18 (8.41)
*Enterobacter agglomerans* (n= 27)	3 (11.1)	1 (3.70)	2 (7.41)
*Enterobacter cloacae* (n= 11)	2 (18.18)	2 (18.18)	1 (9.09)
*Klebsiella *spp. (n = 199)	53 (26.63)	14 (7.04)	28 (14.1)
*Proteus *spp. (n= 3)	0	0	0
Total (n= 455)	114 (25.05)	24 (5.27)	49 (10.77)

As shown in Table 4, analysis of the resistance pattern by gender revealed females had the highest level of MDR 103 (25.88%) and PDR 46 (11.56%). Various resistance patterns were observed across age categories. Individuals below 18 years of age showed the highest XDR (3, 10.71%). The highest MDR and PDR were shown by individuals over 60 years of age; (26, 30.23% and 16, 18.60% respectively) (Table 4). However no statistical differences were observed across resistance categories for gender (X^2^= 3.903, p=0.142) and across age categories (X^2^= 5.188, p=0.520). 

**Table 4:** Categorization of gender and age categories according to antibiotic resistance. MDR (multidrug resistance): non-susceptible to ≥ 1 agent in ≥ 3 antimicrobial categories; XDR (extensive drug resistance): non-susceptible to ≥ 1 agent in all but ≤ 2 categories; PDR (pan-drug resistance): non-susceptible to all antimicrobial categories.

	Category of antibiotic resistance		
Gender	MDR n (%)	XDR n (%)	PDR n (%)
Female (n =398)	103 (25.88)	19 (4.77)	46 (11.56)
Male (n= 57)	11 (19.30)	5 (8.77)	3 (5.26)
Age categories			
Below 18 years (n= 28)	6 (21.42)	3 (10.71)	5 (17.86)
18-34 years (n= 180)	46 (25.56)	7 (3.89)	14 (7.78)
35-60 years (n= 161)	36 (22.36)	7 (4.35)	14 (8.70)
Above 60 years (n= 86)	26 (30.23)	7 (8.14)	16 (18.60)

## Discussion

Our study aimed to investigate the antimicrobial susceptibility patterns of Enterobacteriaceae isolated from UTI outpatients, with a focus on aligning treatment protocols with current resistance trends. The prevalence of UTI was 19.2% (455 out of 2371), with females being the most predominant. Overall, the Enterobacteriaceae uropathogens were proportionately distributed amongst gender and age categories. Organisms exhibited the most resistance to tetracycline (89.5%) and the least resistance to meropenem (16.7%) and ceftriaxone (17.9%). More than a quarter (25.88%) of the organisms exhibited MDR to the studied antibiotics. 

The prevalence of UTIs in the current study is lower than the ones previously conducted in Uganda. In Bushenyi District, the prevalence was 22.3% (67/300) [9] and 86/267 (32.2%) [10]. In Gulu, Odongo and colleagues reported a prevalence of 24.2% (82/339) [11]. In Mulago, Kabugo and colleagues earlier found the prevalence of UTI in adults at 38.8% (54/139) in a Mulago hospital [12]. The higher prevalence of UTIs in the previous studies may be attributed to smaller sample sizes and the inclusion of inpatients, who are usually prone to UTIs. In a recent study conducted in Kenyan health centers on symptomatic patients suggestive of UTI, the prevalence is nearly three times [13] that of our current study. In the Kenyan study, the participants were from urban centers, and samples were collected from several health facilities. These factors together might have contributed to the larger prevalence when compared to our current study. 

The current study corroborates the findings from previous studies that reported a higher prevalence of UTIs in females than males [13,14]. The high prevalence among females in this study may be attributed to the difference in the anatomies of the male and female genitalia, with the female urethra's proximity to the anal orifice. In addition, *E. coli* was most predominant in females, a fact that can be attributed to its ability to move from the perineum areas contaminated with fecal microbes to the moist warm environment of the female genitalia [15]. 

Overall, organisms showed the highest resistance to tetracycline, amoxicillin-clavulanic acid, and amoxicillin, a fact that could be attributed to community members’ tendency to self-medicate with these readily available and relatively cheaper drugs bought from drug shops and pharmacies. Enterobacteriaceae have been reported to be naturally resistant to tetracycline, especially *Enterobacter *spp. [16,17]. Moreover, in our current study, all samples with* Enterobacter agglomerans* were resistant to tetracycline. Surprisingly, resistance to ciprofloxacin, a first choice of treatment of UTIs in Uganda as recommended by Ministry of Health guidelines [18] was low at 24.4 % in our study. This might be a result of the increased use of newer-generation quinolones with a broader spectrum and effectiveness as described elsewhere [19]. Our study also revealed that organisms were less resistant to cefixime and carbapenems. This could be attributed to the fact that these drugs are not the first choice of treatment as per national treatment guidelines and are expensive for community members to use for the treatment of UTIs. These results collectively imply a change in prescription habits and to enhance antibiotic stewardship to include strict use of antibiotics with low organism resistance to avoid the development of further resistance.

It has been well documented that *E. coli *and *Klebsiella *spp., the most common uropathogens, are also the most resistant to a number of commonly used antibiotics for the treatment of UTIs, a finding similar to our study [4,20]. In addition, all studied species showed resistance to a number of commonly used antibiotics with the organisms exhibiting, MDR, XDR, and PDR, an observation reported in earlier studies [14,21,22]. This is an indication that these species have developed resistance mechanisms against a broad spectrum of antibiotics, making infections caused by these species difficult to treat, thereby reducing the treatment options. An earlier study by Maldonado-Barragán and colleagues revealed a predominance of MDR bacteria causing UTIs among symptomatic patients in East Africa [23]. The same study showed that *E. coli *and *Klebsiella *spp. were the predominant MDR organisms [23], as also revealed in the current study. However, a higher prevalence of MDR of 50.9% was reported in Maldonado-Barragán and colleagues’ study when compared to our study, which showed an MDR prevalence of 25.05%. This could be due to the fact that several organisms other than Enterobacteriaceae were studied in the study, with *Staphylococcus aureus *being responsible for most of the MDR; *S. aureus *was not studied in our study. In addition, there were in-patients in Maldonado-Barragán and colleagues’ study, with a likelihood of having acquired hospital organisms, which have been reported to be resistant to several antibiotics [24-26]. 

In our current study, females predominantly harbored organisms that exhibited multiple resistance to the antibiotics, a fact that can be attributed to better health-seeking behaviors in women than men [27], resulting in several prescriptions, thus increasing exposure of Enterobacteriaceae to antibiotics. However, the lack of significant gender-based differences in the proportions of organisms resistant to studied antibiotics could be attributed to the spread of organisms amongst both genders for example through the sharing of toilets, poor health habits, and poor sanitation. It was observed that participants aged 18 to 60 years were the most affected by organisms that demonstrated resistance to many antibiotics, a finding shared by a study conducted in Ghana by Deku and colleagues [14]. Both studies had comparable gender and age distributions of study participants, with females and individuals between 20 and 60 years as the majority. 

As emphasized earlier in recent studies, the development of MDR in low-income regions, for example, Uganda, is attributed to the inappropriate use of antibiotics, a key driver of AMR. The inappropriate use of antibiotics is largely due to two main factors: i) the scarcity of antimicrobial susceptibility data in the region, hampering the use of appropriate empirical treatment for UTIs; and (ii) self-prescription and unrestricted access to antibiotics over-the-counter in the community [28-30].

Furthermore, the presence of PDR in *E. coli*, *Klebsiella *spp., and *Enterobacter *spp. is a worrying finding in our recent study. As PDR indicated resistance to all our studied antibiotic categories, it means the PDR organisms pose the biggest threat to public health as it leaves limited treatment options, highlighting a critical need for alternative treatment approaches, which are currently not readily available. These findings collectively demonstrate the escalating issue of antibiotic resistance across Enterobacteriaceae. This emphasizes the need for vigilant antibiotic stewardship [14]. This therefore emphasizes the need for further research for continuous surveillance of uropathogen susceptibility patterns in several health centers in the region. In addition, robust, advanced, and more accurate methods with automation should be adopted for organism identification and detection of resistance genes in uropathogenic Enterobacteriaceae. The establishment of resistance profiles will guide policy evaluations to include empirical antibiotic prescription guidelines in the management of UTIs caused by uropathogenic Enterobacteriaceae. 

Our study was limited by the smaller number of antibiotics studied. However, the antimicrobial categories were sufficient to meet the study objective**.**

## Conclusions

The prevalence of UTI due to Enterobacteriaceae was relatively low with females predominantly affected. Resistance was across all studied antibiotics, with the least resistance observed for meropenem and ceftriaxone. Organisms exhibited mostly MDR, with *Klebsiella *spp. showing the highest PDR. Our study revealed that the studied uropathogenic Enterobacteriaceae were resistant to the locally and commonly used antibiotics, which implies a serious public health threat. These results not only imply changes in prescription habits and enhancement of antibiotic stewardship including strict use of antibiotics, but also emphasize the need for larger, multiple-center studies with continuous surveillance of uropathogen susceptibility patterns and policy evaluations to guide empirical antibiotic prescription guidelines.
